# Improving the Performance of Mortar under Carbonization Curing by Adjusting the Composition of Ternary Binders

**DOI:** 10.3390/ma17205037

**Published:** 2024-10-15

**Authors:** Fufei Wu, Bumeng Yang, Pengfei Luo, Shuangkuai Dong, Hongying Wang, Qiuyue Zhang, Zonghui Huang, Jun Jiang, Yang Cai, Shan Yang, Fajun Xu

**Affiliations:** Guizhou Normal University, Guiyang 550025, China; 201608014@gznu.edu.cn (F.W.);

**Keywords:** cement, calcium carbide slag, sintered red mud, mechanical parameter, durability, microstructure, carbon sequestration

## Abstract

As the most widely used building material, cement has attracted the attention of scholars because of its large carbon emission. To alleviate the problems of carbon emission and limited resource use caused by cement production, this study focuses on the performance of mortar after carbonization curing by regulating the composition of ternary binders. Testing involved mechanical parameters, carbon shrinkage, water absorption, hydration product, microstructure, adsorption of carbon dioxide, calcium carbonate content, and carbonization degree of mortar, as well as comparisons with the effect of calcium carbide slag and sintered red mud. We carried out several studies which demonstrated that carbonization curing and adjusting the content of calcium carbide slag and sintered red mud were beneficial to improve the mechanical properties, peak load displacement, slope, elastic energy, plastic energy, carbon shrinkage, carbon dioxide adsorption, calcium carbonate content, and carbonization degree of mortar, while the addition of calcium carbide slag and sintered red mud increased the water absorption of mortar, and the greater the dosage, the greater the water absorption. Meanwhile, adding 25%–50% calcium carbide slag and sintered red mud still had negative effects on the mechanical properties of mortar. But carbonation curing and the addition of calcium carbide slag and sintered red mud could promote the hydration reaction and consume calcium hydroxide formed by hydration to form calcium carbonate. When the dosage was 50%, the carbon dioxide adsorption capacity, calcium carbonate content, and carbonization degree of calcium carbide slag mortar were higher than those of sintered red mud mortar, which increased by 29.56%, 102.73%, and 28.84%, respectively. By comparison, calcium carbide slag and sintered red mud still showed superior carbon sequestration capacity, which was higher than fly ash and Bayer red mud. From the experiment, we came to realize that adjusting the composition of cementitious materials could realize the carbon sequestration of cement-based materials and promote the road toward low-carbon sustainable development of cement.

## 1. Introduction

As global awareness of sustainable development and environmental protection rises, environmentally friendly and energy-saving solutions are increasingly being sought by the construction materials industry [1]. Portland cement, as a basic material for the construction industry, has been the focus of industry attention for its high energy consumption and emissions during production [2]. According to statistics, cement production accounts for about 5% to 7% of global CO_2_ emissions [3]. Reducing the use of cement not only reduces energy consumption but also significantly reduces greenhouse gas emissions. Therefore, it is of great practical significance to study how to reduce the use of cement through alternative materials while maintaining or improving the performance of concrete. As industrial byproducts, the effective utilization of calcium carbide slag [4] and sintered red mud [5] not only reduces environmental pollution but also replaces cement to a certain extent, consequently lowering the production costs of concrete. Therefore, exploring the use of calcium carbide slag and sintered red mud in the ternary composite system is conducive to advancing and fostering sustainable development within the cement industry.

Calcium carbide slag is an alkali solid waste produced from calcium carbide hydrolysis for acetylene gas production [6]. Usually, 1.2–1.8 tons of calcium carbide slag are generated when creating 1 ton of acetylene gas [7]. China is recognized worldwide as the foremost producer of calcium carbide slag, with emissions surpassing 5.6 million tons annually [8]. Nevertheless, the comprehensive utilization rate of calcium carbide slag is under 30% [9], and the rest is piled and landfilled in people’s living environments, which causes alarming environmental threats. Its main components are calcium hydroxide and calcium oxide, which can usually be used instead of cement or lime to produce construction materials [10], improve soil [11], capture CO_2_ [12,13], and other applications [14]. Hao et al. [15] mixed 15% and 30% calcium carbide slag into III fly ash and found that β-C_2_S was formed on the surfaces of III fly ash particles when the content of calcium carbide slag was 30%, which increased compressive strength by 21.2% at 28 days and reduced autogenous shrinkage by 30.5% at 28 days. Hanjitsuwan et al. [16] mixed 10%, 20%, and 30% calcium carbide slag into lignite coal fly ash and found that calcium carbide slag could activate ground granulated blast-furnace slag (GGBFS), which could refine microstructures and enhance the strength of alkali-activated GGBFS, suggesting that the optimal content of calcium carbide slag was also 30%. Wang et al. [17] found an increase in carbide slag dosage significantly reduced the plastic viscosity, shear stress, and yield stress of activated ultra-fine-ground granulated blast furnace slag pastes. Generally speaking, it is technically feasible to use carbide slag as a supplementary cementitious material for mortar and concrete production.

Red mud can be divided into Bayer red mud, sintered red mud, and combined red mud according to the production process of aluminum [18]. Usually, 0.8–1.5 tons of red mud is generated when creating 1 ton of alumina, and the accumulated output in China can reach 200 million tons [19]. As for the comprehensive utilization of red mud, it mainly focuses on three aspects. First, alkali, alumina, iron, aluminum, sodium, yttrium, lanthanum, titanium, and vanadium in red mud are recovered [20,21,22,23]. Secondly, red mud is reused to produce building materials, such as cement, bricks, concrete, road base material, and filling material in mining [18,24]. Thirdly, red mud is used to adsorb heavy metal ions (Cu^2+^, Cd^2+^, Pb^2+^), nonheavy metal ions, and molecules in water [25,26]. Meanwhile, it can also absorb heavy metal ions (Cu^2+^, Cd^2+^, Pb^2+^) in the soil [27] and SO_2_ in the waste gas [28]. The comprehensive utilization rate of red mud is under 10% [29]. Generally, the fine particles of Bayer red mud are finer and more dispersed, but sintered red mud has higher strength. Thereby, sintered red mud, distinct from Bayer and combined red muds, can also be derived through the calcination of Bayer red mud. Babisk et al. [30] thought that the corresponding ceramics fired by sintered red mud at 850 °C, 950 °C, and 1050 °C could be used as bricks and roof tiles. Moya et al. [31] proposed a method of activating red mud at high temperatures and found that red mud can be used as the inner and outer surfaces of buildings when the temperature reaches 1140 °C. Ribeiro et al. [32] prepared Brazilian yellow clay by adding 60% red mud and found that it could impair linear shrinkage. Therefore, the comprehensive utilization of red mud can be promoted by calcining Bayer red mud at high temperatures or sintered red mud to prepare cementitious materials.

Carbonation curing enhances the performance of cement-based materials, particularly when admixtures like steel slag, fly ash, red mud, etc., are incorporated. Zhang et al. [33] reviewed the research status of the carbonation curing process and put forward the value potential of recycling industrial waste into building raw materials by carbonation curing. Zhang et al. [34] found that early carbonization curing can improve the carbon fixation ability, mechanical ability, and durability of engineered cementitious composites. Chen et al. [35] put forward a carbonization treatment method for fly ash, and it was found that, upon carbonization, the stability of harmful residues was enhanced, and CO_2_ sequestration was facilitated. Wang et al. [36] proposed to improve the carbon fixation capacity of red mud by adjusting the pressure of CO_2_ gas. However, it is worth noting that after carbonization curing, the alkalinity of cementitious materials will be reduced. The greater the admixture content, the greater the decrease in alkalinity. Meanwhile, the carbonation curing process can consume calcium hydroxide after cement hydration and also reduce the alkalinity of cementitious materials. Whether the addition of alkaline admixture can make up for this defect deserves further in-depth study.

In our work, a sustainable cementitious material containing calcium carbide slag and sintered red mud was proposed for the first time. Primarily, the compressive strength of mortar after carbonation curing was tested by YAW-300B, and then their compression toughness was analyzed. Secondly, the carbon shrinkage, mass loss and water absorption of mortar were tested. Furthermore, the microstructure and elements of the hydration products of mortar were analyzed by Zeiss scanner. Finally, the amount of calcium hydroxide (CH), calcium silicate hydrate (CSH), and calcium carbonate (CaCO_3_) was calculated after high-temperature calcination, and the carbon sequestration properties of ternary cementitious materials were calculated, such as carbon dioxide adsorption and carbonation degree. Meanwhile, the influence mechanism of calcium carbide slag and sintered red mud on the carbon sequestration properties of mortar was revealed. These studies are helpful in understanding the influence trend of alkaline admixture on the carbon sequestration properties of mortar and also present a novel perspective for the utilization of alkaline admixtures.

## 2. Materials and Methods

### 2.1. Experimental Materials

Ordinary Portland cement (OPC, Grade 42.5 R according to Chinese national standard GB175–2023 [37]), calcium carbide slag (CCS), and sintered red mud (SRM) were employed in this experiment. Their chemical compositions are given in Table 1. The mixing and curing water were laboratory tap water. High-silicon content standard sand came from Xia Men ISO standard sand Co., Ltd. of China (Xiamen, China), and its particle sizes are 0.08–2.0 mm, with a water absorption rate of 0.12%.

### 2.2. Experimental Procedures

**Figure 1:**
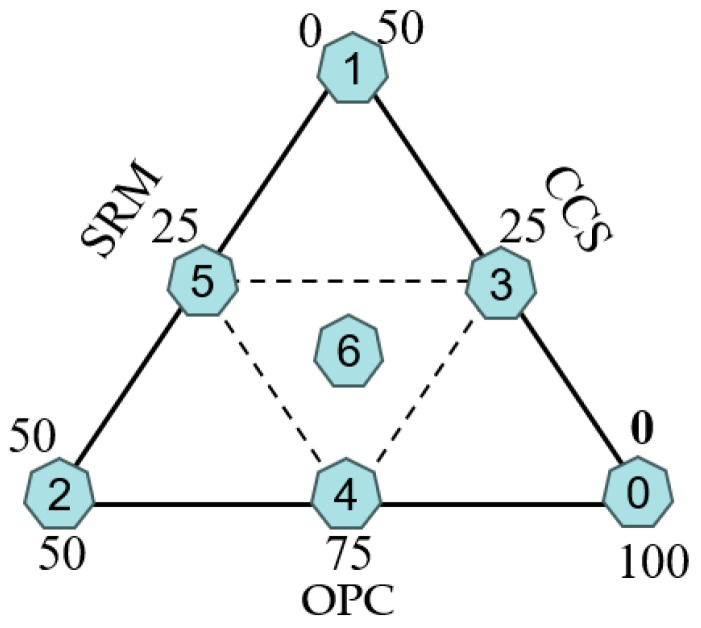
Factor design of cementitious materials.

**Figure 2:**
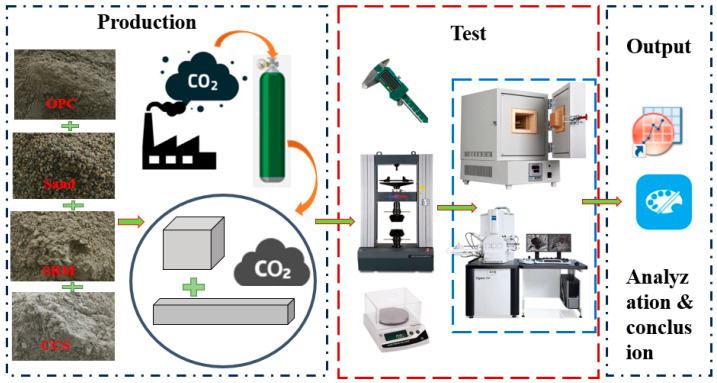
The flow chart of research.

The series of mortar mixtures with ternary binders containing ordinary Portland cement, calcium carbide slag, and sintered red mud were designed in accordance with the factor designing method as stated in reference [38]. The water-to-binder ratios of all the mixes were fixed at 0.4. Seven mortar mixtures, projected in Figure 1 and given in Table 2, after stirring evenly, were put into a cube test mold with a size of 40 mm × 40 mm × 40 mm, and they were placed in an environment with humidity no less than 95%, temperature of 20 ± 2 °C, CO_2_ concentration of 99.8% and pressure of 1Mpa. After curing for 3 days and 28 days, the mechanical properties, shrinkage properties, and hydration products of the mortar were evaluated. The flow chart of the research is shown in Figure 2.

### 2.3. Experimental Methods

The compressive strength of seven groups of mortar mixture samples was evaluated with a YAW-300B (produced by Jinan Times Gold Testing Machine Co., Ltd., Jinan, China) at 3 days and 28 days curing periods, and each group had 3 specimens with a size of 40 mm × 40 mm × 40 mm. This evaluation process was based on the guidelines outlined in the GB/T 17671-2021 standard [39], and their loading rate was 2 mm/min, ensuring that the complete stress–strain curve could be obtained smoothly.

Seven groups of prism specimens with a size of 25 mm × 25 mm × 285 mm were employed to evaluate the shrinkage properties of ternary binders using a 300 mm Stainless Steel Digital Caliper (MNT919030). After all of the samples were demolded, the specimens were put in a 99.8% CO_2_ environment, with a temperature of 20 °C and relative humidity of 60% [40], while evaluating the carbon shrinkages of ternary binders.

After 28 days of carbonization curing, 7 samples with smooth surfaces were obtained after cutting, and then they were immersed in alcohol for 48 h to stop hydration and tested by Zeiss scanner for the morphology of hydration products.

Different hydration products were calculated according to the mass loss after different high-temperature calcinations [41,42]. For each sample, about 20 ± 1 g of paste power was heated from temperature to 950 °C, holding calcination for 1 h at each calculated temperature. The amount of CSH ($W_{CSH}$), CH ($W_{CH}$), absorption of carbon dioxide ($W_{{CO}_{2}}$), calcium carbonate ($W_{{CaCO}_{3}}$), and carbonation degree (CD) was calculated with Equations (1)–(6) [36,41,42,43].

(1) $$W_{CSH}\;(wt\%)=\frac{m_{105}-m_{420}}{m_{105}}\times 100$$

(2) $$W_{CH}\left(wt\%\right)=4.12\times \frac{m_{420}-m_{550}}{m_{105}}\times 100$$

(3) $$W_{{CO}_{2}}\left(wt\%\right)=\frac{m_{550}-m_{950}}{m_{105}}\times 100$$

(4) $$W_{{CaCO}_{3}}\left(wt\%\right)=2.27\times \frac{m_{550}-m_{950}}{m_{105}}\times 100$$

(5) $${TH}_{{CO}_{2}}\left(wt\%\right)=0.785\times \left(CaO-0.7{SO}_{3}\right)+1.09{Na}_{2}O+0.93K_{2}O$$

(6) $$CD\left(wt\%\right)=\frac{{CO}_{2}\left(wt\%\right)}{95\%\times {TH}_{{CO}_{2}}}\times 100$$

where *M*_105_, *M*_420_, *M*_550_, *M*_950_, were mass of pastes at 105 °C, 420 °C, 550 °C, and 950 °C, respectively. CaO, SO_3_, Na_2_O, and K_2_O composed the cementitious materials. ${TH}_{{CO}_{2}}$ is the maximum theoretical CO_2_ uptake of cement.

## 3. Results and Discussion

### 3.1. Compressive Strength

**Figure 3:**
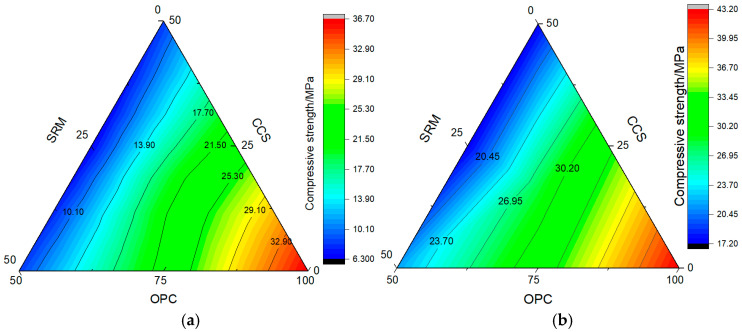
Compressive strength of mortar: (**a**) carbonization curing for 3 d; (**b**) carbonization curing for 28 d.

Analyses of the mechanical properties of ternary binders have been conducted by several scholars. However, ternary binders were prepared with different amounts of calcium carbide slag and sintered red mud for first time in this paper. Secondly, the chemical composition of calcium carbide slag and sintered red mud varies considerably around the world, necessitating research to be conducted on these materials from diverse locations. In addition, the effects of fixed CO_2_ pressure and curing time on the mechanical properties of ternary binders were investigated. In order to further clarify the influence of calcium carbide slag and sintered red mud content and CO_2_ curing time on the mechanical properties of ternary binders, the results are shown in Figure 3.

As can be seen from Figure 3, under constant CO_2_ pressure curing, the content and combination ratio of calcium carbide slag and sintered red mud can affect the mechanical properties of ternary binders. Without CO_2_ curing, the compressive strength of cement mortar was 33.93 MPa at 3 days. It was promoted by 8.14% after CO_2_ curing for 3 days, and similar results were found in reference [2] and reference [5]. It is worth noting that even under carbonation curing at 3 days, the mechanical properties of mortar samples could be reduced when 25% calcium carbide slag or 25% sintered red mud was mixed, and the greater the content, the greater the reduction in mechanical properties: it was reduced by 63.7% for 50% calcium carbide slag and 59.5% for 50% sintered red mud. Comparing the influence of the content of calcium carbide slag and sintered red mud on the compressive strength of mortar, it was found that the mechanical properties of mortar mixed with calcium carbide slag were higher than those of sintered red mud mortar, and even when the content was 50%, this trend remained consistent. Reference [2] and reference [5] also showed that the greater the content of Bayer red mud, phosphogypsum, and lithium slag, the greater the mechanical properties. In addition, after 33.3% calcium carbide slag and sintered red mud were compounded, the compressive strength of the mortar was 11.4% lower than that of 25% calcium carbide slag mortar and 8.5% lower than that of sintered red mud mortar. Therefore, the mechanical properties of calcium carbide slag combined with sintered red mud are better than those of single mixing, but the same trend does not exist when the composite content reaches 50%.

After carbonization curing for 28 d, the compressive strengths of the seven groups improved by varying degrees. At a dosage of 25%, the compressive strength of the carbide slag group was 32.95 MPa, which was 42.33% higher than that of carbonization curing for 3 days, while the compressive strength of the sintered red mud group was 31.97 MPa lower than that of the carbide slag group, and its growth rate was 0.4% higher than that of the carbide slag group. At a dosage of 50%, the compressive strength and growth range of the carbide slag group were 21.6 MPa and 157.1%, respectively, which were higher than those of the sintered red mud group, while the compressive strength of the composite group at a dosage of 50% increased by 171.0%, which was 133.3% higher than when the dosage was 33.4%. Therefore, adjusting the composition of the admixture is beneficial to the role of carbonization and promotes the improvement of mechanical properties.

### 3.2. Compression Toughness

**Figure 4:**
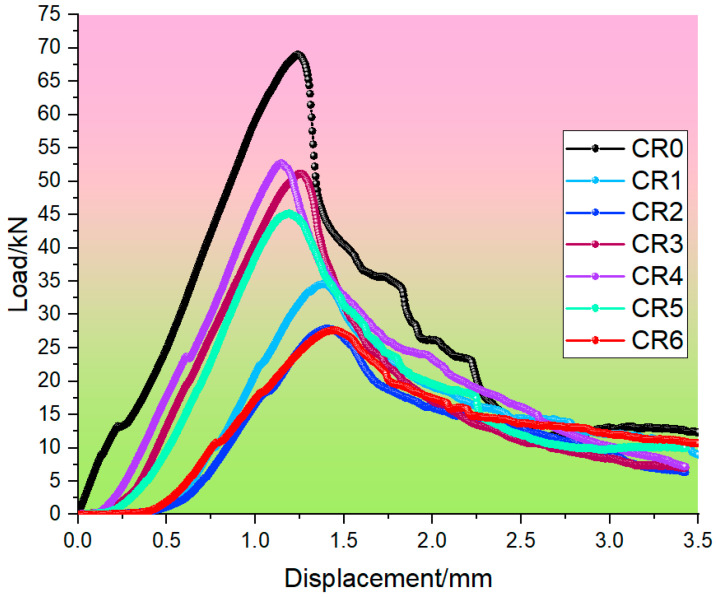
Load vs. displacement curves of mortar.

**Figure 5:**
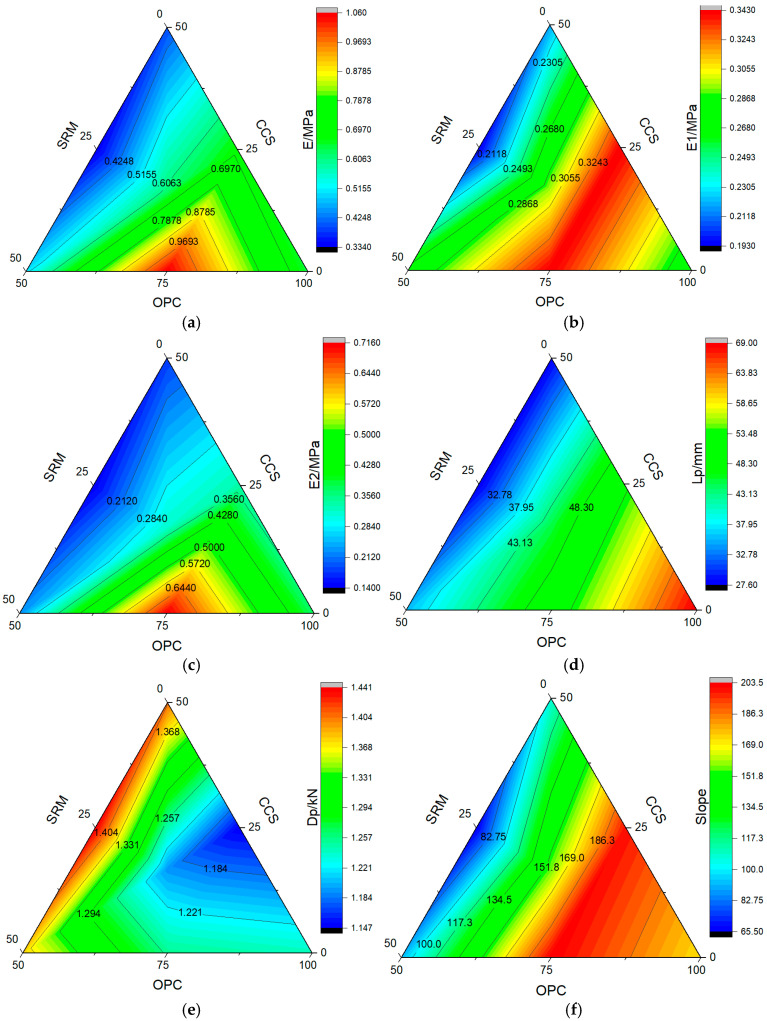
Mechanical parameters of mortar: (**a**) *E*; (**b**) *E*1; (**c**) *E*2; (**d**) *Lp*; (**e**) *Dp*; (**f**) slope.

The load vs. displacement curves of seven mortar groups at carbonization curing for 28 days are shown in Figure 4. All mechanical parameters shown in Figure 5, such as peak load displacement (*Dp*), peak load (*Lp*), slope, total (*E*), elastic (*E*1), and plastic energy (*E*2), are average values of three samples per mix.

The peak strain on the load–displacement curve, corresponding to the peak stress, represents the mortar specimen’s peak strain, which can well reflect the ductility characteristics of mortar, and the greater the peak strain of the mortar specimen, the greater its ductility [44]. The peak strain of mortar at carbonization curing for 28 days is shown in Figure 5. Without CO_2_ curing, the peak load displacement of cement mortar was 1.242 mm. Wang et al. [43] found that after adding Nano-MgO-modified cement soil, the peak load displacement reached up to 4 mm, while Anjaneya et al. [45] found that it was 0.674 mm for ordinary Portland cement CEM-I 52.5 N. Analogously, carbonization curing also can increase peak load displacement, but it is not obvious. It did not exceed 1% in the paper, compared with standard curing. This shows that the cement type also affects the peak displacement of mortar, maintenance method, type, and dosage of admixture. As can be seen from Figure 5, after adding calcium carbide slag and sintered red mud, the peak load displacement of mortar constantly changes. When the individual dosage was 25%, or when the composite dosage was 33.3%, the ductility characteristics of mortar were all poor compared with the reference mix. However, the ductility characteristics of cement mortar were better than those of cement mortar, whether the single dosage was 50% or the composite dosage was 50%. Meanwhile, it was readily apparent that calcium carbide slag had a more significant contribution to the ductility characteristics of mortar than sintered red mud. This is primarily attributed to the differences in alkalinity and activity between the admixtures, which subsequently compensate for deficiencies in ductility.

Elastic modulus reflects the rigidity of mortar and is widely used in many fields, such as engineering, construction, and manufacturing. The greater the elastic modulus, the more the rigidity of the material can be stretched or compressed. Conversely, the smaller the elastic modulus, the more easily the material can be deformed [46]. Without CO_2_ curing, the elastic energy (*E*1) and plastic energy (*E*2) of cement mortar were 0.137 MPa and 0.312 MPa, and they were 0.353 MPa and 0.320 MPa after carbonization curing for 28 days, as could be seen from Figure 5. Anjaneya et al. [45] found that they were 0.208 MPa and 0.714 MPa for ordinary Portland cement CEM-I 52.5 N. In addition, after adding calcium carbide slag and sintered red mud, the variations in elastic energy and plastic energy in all the mixes with increased dosage are shown in Figure 5. The elastic energy was higher than that of cement mortar without CO_2_ curing, with an increase of 40.86–151.82%. However, it was lower than that of cement mortar with carbonization curing, with a decrease of 2.27–45.33%, which might be ascribed to the lower toughness of admixture compared with cement, as well as the degradation in microstructure compactness due to the use of admixture instead of cement. The difference was that when the content of calcium carbide slag was 25%, the plastic energy was higher than that of cement mortar by 8.01% with standard curing and 5.31% with carbonation curing. The other groups were all lower than cement mortar, and the maximum was less than 54.69%.

The slope of the load vs. the displacement curve after the peak indicates the ductility of the mortar. Without CO_2_ curing, the slope of the cement mortar was 365.95, and it was 171.26 after carbonization curing for 28 days. However, Anjaneya et al. [45] found that it was 657.2 for ordinary Portland cement CEM-I 52.5 N. Therefore, different types of cement, carbonation curing time, and CO_2_ concentration can affect the ductility of mortar, which is mainly due to the hydration process of cement. The faster the hydration reaction, the better the ductility of the mortar. In addition, as can be seen from Figure 5, the curves of all the mixes had different trajectories, suggesting that both calcium carbide slag and sintered red mud had negligible effects on the stiffness of the control mix. After adding calcium carbide slag and sintered red mud, the slope of the mortar was less than that of the cement at standard curing, reduced by 44.50–82.01%, while after adding 25% calcium carbide slag or 25% sintered red mud, the slopes of mortar were higher than those of cement at carbonization curing, an increase of 16.62% and 18.60%, respectively. Relatively speaking, sintered red mud had a greater contribution to the ductility of mortar than that of calcium carbide slag. Additionally, the slope of the remaining groups was lower than that of cement mortar, which was reduced by 8.19–61.55%. This is mainly attributable to the lower activity of the admixture compared with cement, generating a small amount of hydration product in the former to enlarge the weak spots created by the admixture.

### 3.3. Carbon Shrinkage and Mass Loss

**Figure 6:**
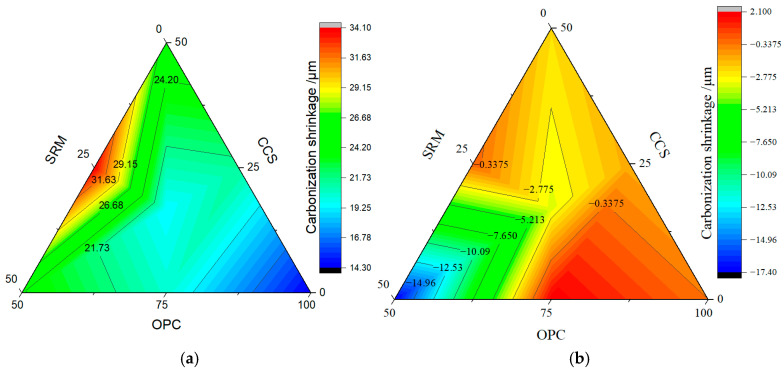
Carbon shrinkage of mortar: (**a**) carbonization curing for 3 d; (**b**) carbonization curing for 28 d.

**Figure 7:**
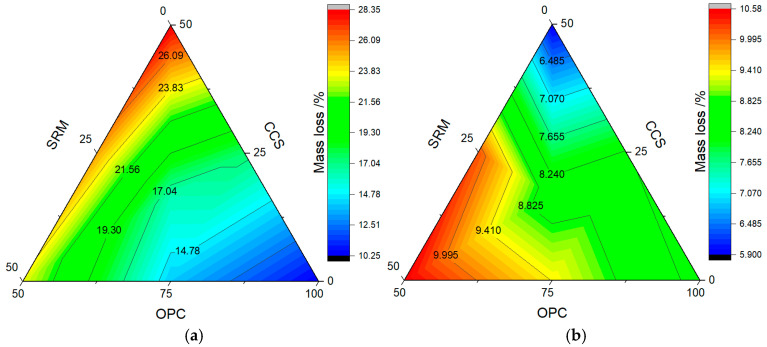
Mass loss of mortar: (**a**) carbonization curing for 3 d; (**b**) carbonization curing for 28 d.

The dry shrinkage specimen was put into a carbonization environment, its shrinkage value was measured as carbonization shrinkage, and its mass loss was also tested. The results from carbon shrinkage tests and mass loss tests are shown in Figure 6 and Figure 7, respectively.

Early cracks in concrete were controlled by shrinkage after concrete solidification. At this stage, the hardened concrete became hard enough to restrain any dimensional adjustment, leading to internal stress and, eventually, cracking [47]. As can be seen from Figure 6, it is obvious from the results that calcium carbide slag, sintered red mud, and carbonization curing were able to inhibit shrinkages in the mortar. The restrain on shrinkage became more protuberant with the increase in calcium carbide slag and sintered red mud content, as well as with the prolongation in carbonation curing age. Generally, the drying shrinkage of pure cement mortar is about 400 μm/m and 600 μm/m after 3 d and 28 d. After adding 25% calcium carbide slag or sintered red mud, the carbonization shrinkage was reduced to 21.3 μm/m or 20.3 μm/m after carbonization curing for 3 days, respectively. However, when the addition amount increased to 50%, the carbonization shrinkage increased to 25.6 μm/m for sintered red mud and 24.3 μm/m for calcium carbide slag. This is mainly attributable to the water loss of calcium carbide slag and sintered red mud in ternary binders. After the carbonation curing age was extended to 28 days, the carbon shrinkages of ternary binders were more prominent, and the phenomenon of expansion appeared. Therefore, it had expanded by 1.33 μm/m for 25% calcium carbide slag, 17.33 μm/m for 50% calcium carbide slag, and 2.33 μm/m for 25% sintered red mud. From these results, carbonation curing is beneficial to inhibit the shrinkage of mortar and reduce the occurrence of early cracks.

Meanwhile, as can be seen from Figure 7, it is evident from the results that the addition of calcium carbide slag and sintered red mud content and carbonation curing can also improve the mass loss of ternary binders. Without CO_2_ curing, the mass loss of cement mortar was 26.0% at 3 days and 26.9% at 28 days. After carbonation curing for 3 days, the mass loss was 28.33% when the content of calcium carbide slag was 25%, which was higher than 26%, and the rest of the groups were lower than 26%, while the mass losses of ternary binders were less than 26.9% after carbonation curing for 28 days. Therefore, carbonation curing is beneficial to the reaction of cementitious materials and promotes the formation of hydration products.

### 3.4. Water Absorption

**Figure 8:**
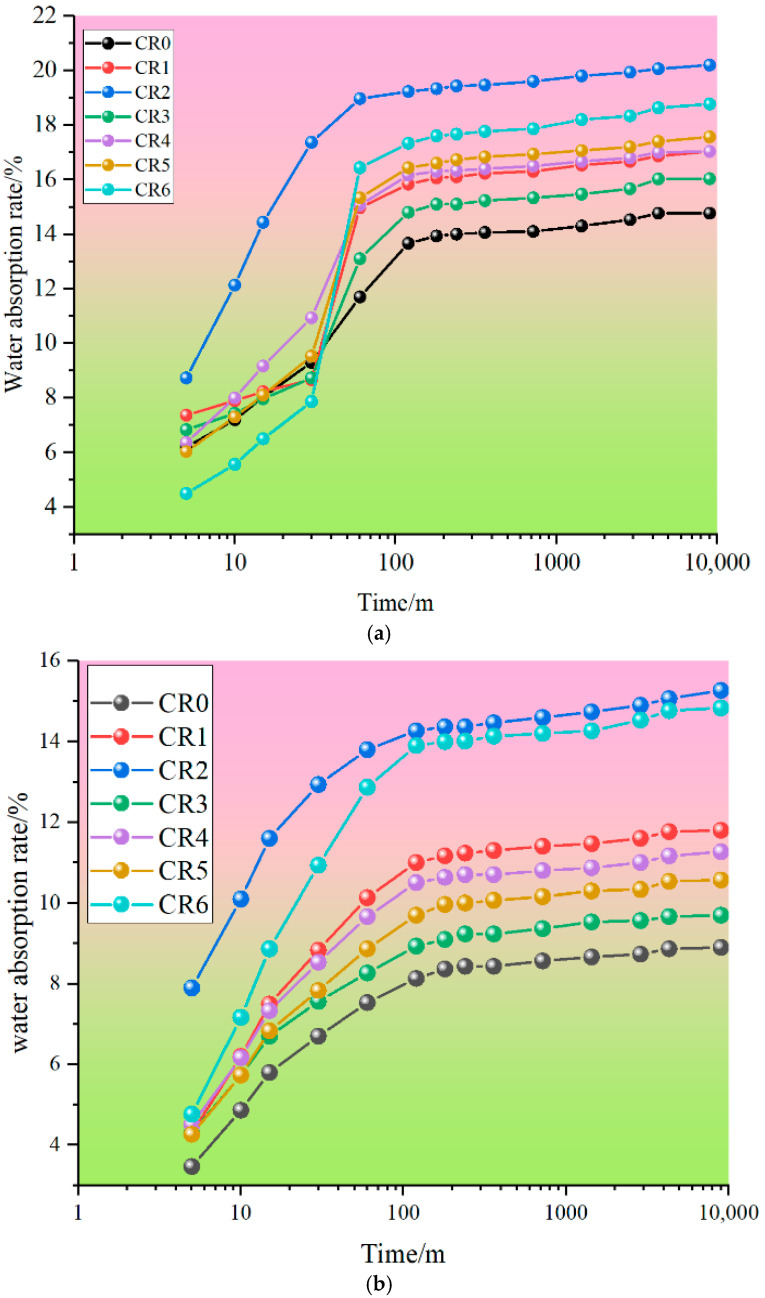
Water absorption rate of mortar: (**a**) carbonization curing for 3 d; (**b**) carbonization curing for 28 d.

**Figure 9:**
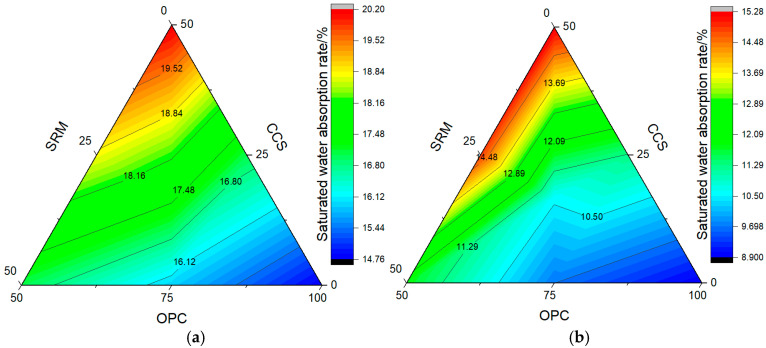
Saturated water absorption rate of mortar: (**a**) carbonization curing for 3 d; (**b**) carbonization curing for 28 d.

The results from water absorption rate tests are shown in Figure 8 and Figure 9. As can be seen from Figure 8, the water absorption rate of mortar containing calcium carbide slag and sintered red mud progressively increased as the dosage was raised, which could be roughly divided into two stages, namely, the rising growth stage and the stable stage, whether it was carbonized for 3 days or 28 days. In addition, when the water absorption time exceeded 100 min, the water absorption rate of mortar containing calcium carbide slag and sintered red mud was higher than that of pure cement mortar. The main reason is that calcium carbide slag and sintered red mud have strong hygroscopicity, and the mixing amount of calcium carbide slag and sintered red mud is large, which reduces the gelling property of ternary cementitious materials; therefore, the water absorption rate of ternary cementitious material is increased.

Without CO_2_ curing, the saturated water absorption rate of cement mortar was 15.4% at 3 days and 9.3% at 28 days. However, after carbonation curing for 3 days and 28 days, the saturated water absorption of cement mortar decreased by 14.77% and 8.90%, which is 4.76% and 4.30% lower than that of normal curing. This also shows that carbonation curing is beneficial to improve the density of cement mortar, which is similar to the mechanical properties. After adding calcium carbide slag and sintered red mud, the saturated water absorption of the mortar was higher than the cement mortar. When the dosage was 25%, the saturated water absorption rate of calcium carbide slag mortar was higher than that of sintered red mud mortar, which was 6.90% at 3 days and 16.15% at 28 days. However, when the content increased to 50%, it showed an opposite trend. At this time, the water absorption of mortar containing calcium carbide slag and sintered red mud was between sintered red mud mortar and calcium carbide slag mortar. This also shows that adjusting the content of calcium carbide slag and sintered red mud is beneficial to improve the water absorption of mortar.

### 3.5. Hydration Products and Microstructure

**Figure 10:**
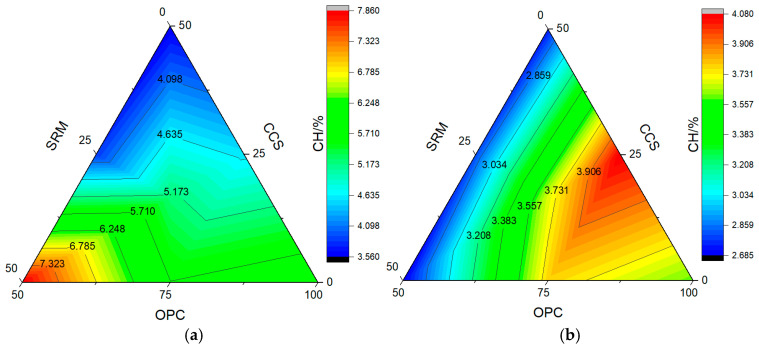
The content of calcium hydroxide: (**a**) carbonization curing for 3 d; (**b**) carbonization curing for 28 d.

**Figure 11:**
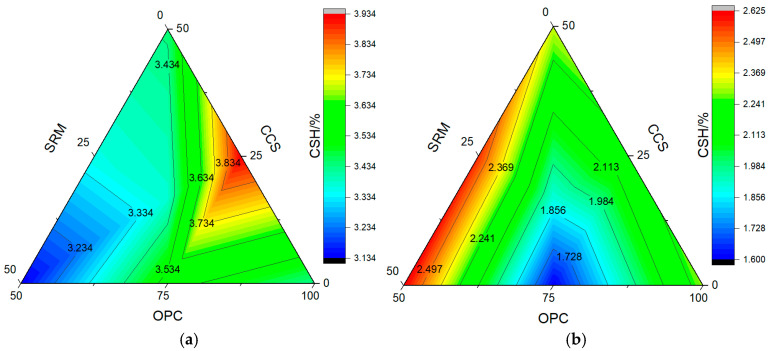
The content of calcium silicate hydrate: (**a**) carbonization curing for 3 d; (**b**) carbonization curing for 28 d.

**Figure 12:**
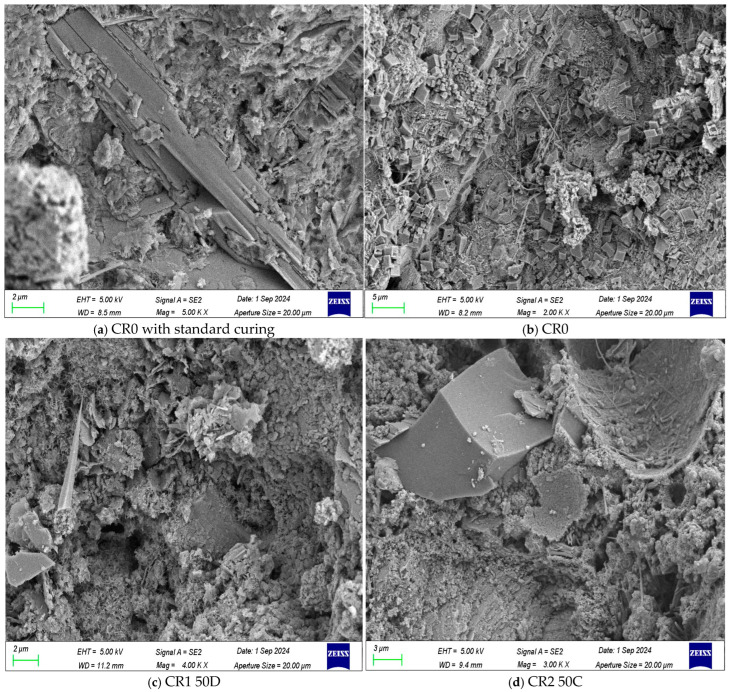

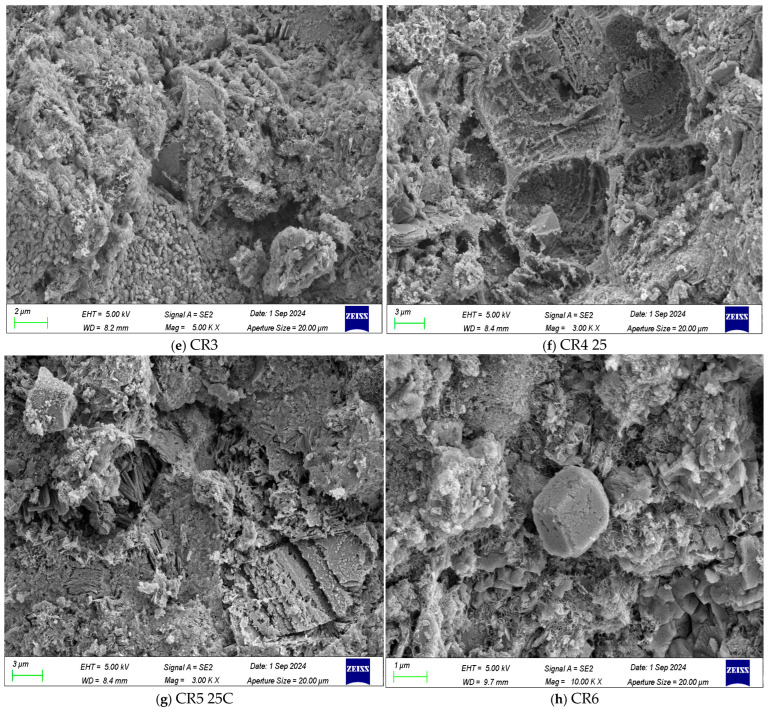
Microstructure of mortar carbonized for 28 days: (**a**) CR0 with standard curing; (**b**) CR0; (**c**) CR1; (**d**) CR2; (**e**) CR3; (**f**) CR4; (**g**) CR5; (**h**) CR6.

After cement hydration, hydration products such as calcium silicate hydrate (CSH), calcium hydroxide (CH), and ettringite (AFt) are usually generated. In this paper, the paste was calcined by a high-temperature calcination method, and the temperature was kept for 1 h. Then, the content of hydration products was calculated by the mass loss at different calcination temperatures. The calculation results of calcium hydroxide and calcium silicate hydrate in paste are shown in Figure 10 and Figure 11, respectively, and the microstructure of mortar at carbonization curing for 28 days is shown in Figure 12.

Without CO_2_ curing, the content of CH in cement paste was 3.55% at 3 days and 4.64% at 28 days, and the content of CSH was 4.70% at 3 days and 4.45% at 28 days. After carbonation curing, the content of CH in cement paste increased to 6.04% at 3 days and reduced to 3.60% at 28 days, and the content of CSH reduced to 3.43% at 3 days and 2.30% at 28 days. This is primarily attributed to the fact that carbonation curing enhances the hydration reaction, resulting in the consumption of CH formed through hydration and the formation of calcium carbonate [48].

As could be seen from Figure 10, when the dosage was 25%, the content of CH in calcium carbide slag paste was less than that of sintered red mud paste, while when the dosage was 50%, there was an opposite trend, and the content of CH decreased significantly than that of cement paste after adding calcium carbide slag and sintered red mud. This shows that adding calcium carbide slag and sintered red mud can also consume CH formed by cement hydration and then form CSH. As can be seen from Figure 11, after adding 25% calcium carbide slag or sintered red mud, the content of CSH was 3.93% in calcium carbide slag paste and 3.56% in sintered red mud paste at carbonation curing for 3 days, while they were 3.13% and 3.44% at carbonation curing for 28 days. This shows that carbonation curing could promote the formation of early-age calcium hydroxide and convert part of CSH into calcium carbonate, which further reduces the content of CSH.

As can be seen from Figure 12a, the microstructure of hydration products was clearly visible, especially needle-like ettringite, plate-like and layered calcium hydroxide, fibrous hydrated calcium silicate, and a small amount of micropores. After carbonation curing for 28 days, the micromorphology of the calcium hydroxide in the mortar disappeared obviously, and the fibrous calcium silicate hydrate also decreased. Meanwhile, the quantity of cubic calcium carbonate increased significantly. This also shows, once again, that carbonization is beneficial to the transformation of calcium hydroxide into calcium carbonate and the enhancement of its compactness, so it is beneficial to the improvement of mechanical properties and durability of mortar. After adding calcium carbide slag and sintered red mud, even under carbonation curing, the microstructure of mortar was still dominated by needle-like ettringite, layered calcium hydroxide, and fibrous hydrated calcium silicate, with a small amount of cubic calcium carbonate and micropores. In addition, it was found that the addition of calcium carbide slag was beneficial to the consumption of calcium hydroxide and the hydration product of cement, and it reduced the amount of reaction with CO_2_, such as CR1, where no obvious cubic calcium carbonate was detected. However, when the content of calcium carbide slag reduced to 25%, as exemplified by CR4, not only cubic calcium carbonate but also layered calcium hydroxide could be observed, which provided the possibility for carbonization reaction in mortar matrix. Analogously, layered calcium hydroxide, fibrous calcium silicate hydrate, and cubic calcium carbonate could still be seen when 33.4% sintered red mud and calcium carbide slag were mixed. However, when the compound dosage was 50%, the kind of hydration products did not change, but the compactness was obviously improved. From the above, it can be seen that adjusting the proportion and dosage of admixture types is beneficial to improving the compactness of the mortar matrix and the content of hydration products.

### 3.6. Carbon Sequestration

**Figure 13:**
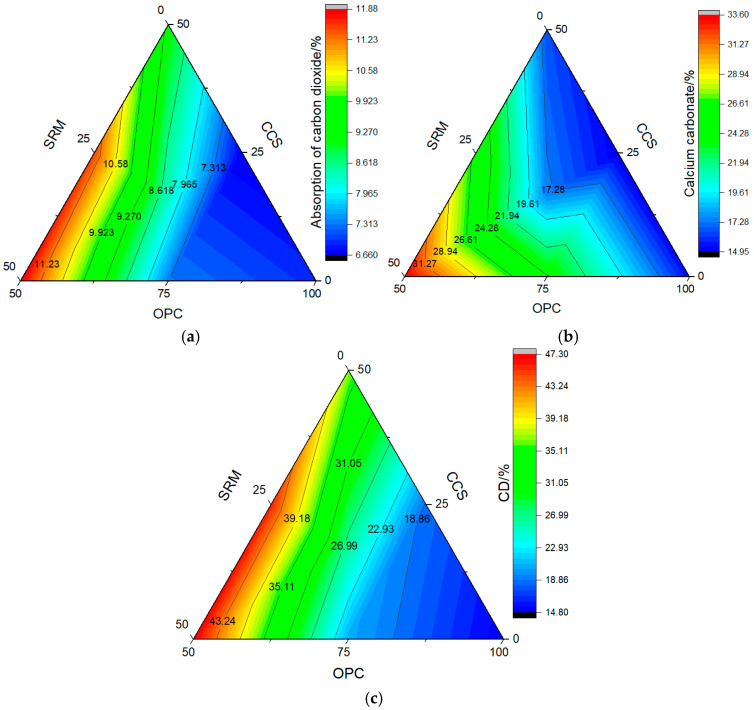
Carbon sequestration of mortar: (**a**) adsorption of carbon dioxide; (**b**) calcium carbonate; (**c**) carbonation degree.

Calcium hydroxide is generated after cement hydration, which can react with CO_2_ to generate calcium carbonate. According to the research results of references [36,41,42,43], carbon fixation can be obtained by high-temperature calcination. In order to explore the change in carbon fixation, such as CO_2_ adsorption rate, calcium carbonate, and carbonization degree, the effects of sintered red mud mortar and calcium carbide slag on the carbon fixation of mortar at carbonization curing for 28 days were investigated, and the results are shown in Figure 13.

As can be seen from Figure 13, the adsorptions of carbon dioxide, calcium carbonate content, and carbonization degree of mortar were 6.931%, 15.362%, and 14.89%, respectively. After adding calcium carbide slag and sintered red mud, the adsorptions of carbon dioxide, calcium carbonate content, and carbonization degree of mortar containing 25% calcium carbide slag were lower than that of cement mortar, and they had decreased by 3.78%, 2.46%, and 23.17%, respectively. These results suggest that calcium carbide slag participates in the secondary hydration reaction of cement, which reduces the content of CH in the mortar matrix. Under varying dosages, the mortar exhibited higher levels of carbon dioxide adsorption, calcium carbonate content, and carbonization compared with cement mortar. Comparatively speaking, when the dosage was 25%, the adsorptions of carbon dioxide, calcium carbonate content, and carbonization degree of mortar containing sintered red mud were higher than that of mortar containing calcium carbide slag, an increase of 10.03%, 62.79%, and 9.82%, respectively. However, when the dosage was 50%, they showed the opposite trend, that is, calcium carbide slag mortar was higher than that of sintered red mud, an increase of 29.56%, 102.73%, and 28.84%, respectively. Even if calcium carbide slag and sintered red mud were compounded, the superposition effect was not fully exerted, and the indexes such as adsorption of carbon dioxide, calcium carbonate content, and carbonization degree were still lower than calcium carbide slag mortar. These indicators show that the carbon sequestration of mortar is related to the composition, alkalinity, and activity of cementitious materials.

**Figure 14:**
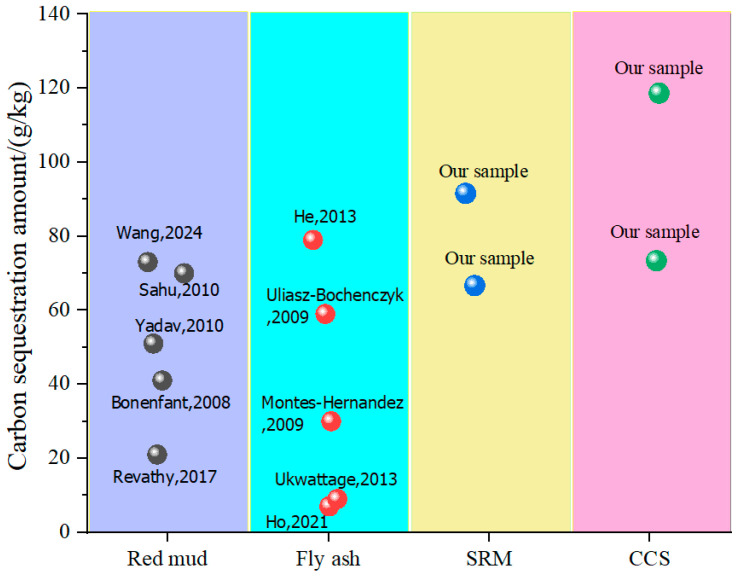
Carbon sequestration amount of various cementing materials [36,48,49,50,51,52,53,54,55,56].

Figure 14 shows the experimental results of carbon sequestration in different references. As can be seen from Figure 14, the experimental results of carbon sequestration were quite different due to different processes. According to references [48,49,50,51,52,53,54,55,56] and our experimental results, calcium carbide slag and sintered red mud showed a good carbon sequestration effect under CO_2_ pressure curing. Generally speaking, carbon sequestration can be achieved by adjusting the composition of cementitious materials under certain external conditions.

## 4. Conclusions

Our work involved studies of mechanical parameters, carbon shrinkage, water absorption, hydration product, microstructure, adsorption of carbon dioxide, calcium carbonate content, and carbonization degree of mortar, and it was extremely beneficial to the low-carbon sustainable development of cement.

(1) The compressive strength of cement mortar was promoted by 8.14% after CO_2_ curing for 3 days; thus, carbonization curing was beneficial to improve the mechanical properties of mortar, but it still had negative effects when adding 25%, 50% calcium carbide slag, and sintered red mud. However, adjusting the content of calcium carbide slag and sintered red mud was beneficial to improve the peak load displacement, slope, and elastic and plastic energy of mortar.
(2) Carbonization curing and the addition of calcium carbide slag and sintered red mud could inhibit the shrinkage of mortar. With an increase in the content of calcium carbide slag and sintered red mud, the inhibition of carbonation curing age on shrinkage was more prominent, and an even swelling phenomenon appeared and reached the maximum of 17.33 μm/m. However, the addition of calcium carbide slag and sintered red mud increased the water absorption of mortar, and the maximum increase was 6.37% at 28 days.
(3) After carbonization curing for 28 days, the content of CH and CSH in cement paste reduced to 3.60% and 2.30%, respectively. Therefore, carbonation curing could promote the hydration reaction and consume CH formed by hydration to form calcium carbonate. A similar rule still existed even if the mixing amount and composition ratio of calcium carbide slag and sintered red mud were adjusted.
(4) Adjusting the content of calcium carbide slag and sintered red mud could increase the adsorption of carbon dioxide, the content of calcium carbonate, and the degree of carbonization. When the dosage was 50%, the carbon dioxide adsorption capacity, calcium carbonate content, and carbonization degree of calcium carbide slag mortar were higher than those of sintered red mud mortar, which increased by 29.56%, 102.73%, and 28.84%, respectively. Even if calcium carbide slag was compounded with sintered red mud, the superposition effect was not fully exerted, and the indexes such as carbon dioxide adsorption capacity, calcium carbonate content, and carbonization degree were still lower than those of carbide slag mortar. However, calcium carbide slag and sintered red mud still showed superior carbon sequestration capacity, which was higher than fly ash and Bayer red mud.

## Figures and Tables

**Table 1:** Chemical composition of OPC, CCS, and CRM.

Name	SiO_2_	Al_2_O_3_	Fe_2_O_3_	CaO	MgO	SO_3_	TiO_2_	Na_2_O	K_2_O	Others
OPC/wt.%	21.58	6.78	4.77	60.54	2.81	2.36	0	0.12	0.21	0.83
CCS/wt.%	1.55	1.96	0.48	67.87	0	0.31	0	2.52	1.88	23.43
SRM/wt.%	23.43	6.42	9.37	48.39	1.97	0.11	3.45	1.63	2.05	3.18

**Table 2:** Composition of cementitious materials (g).

No.	OPC	CCS	SRM	Water	Sand
CR 0	450	0	0	180	450
CR 1	225	0	225	180	450
CR 2	225	225	0	180	450
CR 3	337.5	0	112.5	180	450
CR 4	337.5	112.5	0	180	450
CR 5	299.7	75.15	75.15	180	450
CR 6	225	112.5	112.5	180	450

## Data Availability

The original contributions presented in the study are included in the article, further inquiries can be directed to the corresponding author.

## References

[1] Liu Z., Guo A. (2021). Application of Green Building Materials and Multi-objective Energy-Saving Optimization. Int. J. Heat Technol..

[2] Dong S., Yu S., Chen L., Zhuo Q., Wu F., Xie L., Liu L. (2023). Effects of pretreated phosphogypsum and granulated blast-furnace slag on the rheological properties of the paste excited by NaOH. Molecules.

[3] Cheng D., Reiner D.M., Yang F., Cui C., Meng J., Shan Y., Liu Y., Tao S., Guan D. (2023). Projecting future carbon emissions from cement production in developing countries. Nat. Commun..

[4] Pang H., Wei Q., Xu Y., Zhang Y., Xu D., Liu J., He J., Lu J. (2024). Two wastes into one resource: Carbide slag-driven anaerobic fermentation of excess sludge towards short-chain fatty acids recovery. Chem. Eng. J..

[5] Dong S., Zhuo Q., Chen L., Wu F., Xie L. (2023). Reuse of pretreated red mud and phosphogypsum as supplementary cementitious material. Sustainability.

[6] Gong X., Zhang T., Zhang J., Wang Z., Liu J., Cao J., Wang C. (2022). Recycling and utilization of calcium carbide slag—Current status and new opportunities. Renew. Sustain. Energy Rev..

[7] Altiner M. (2019). Use of taguchi approach for synthesis of calcite particles from calcium carbide slag for CO_2_ fixation by accelerated mineral carbonation. Arab. J. Chem..

[8] Li M., Tan H., Zhang J., XDeng Kong X., Chen P., Jian S., He X., Yang J. (2023). Enhancement in compressive strength of carbide slag activated ground granulated blast furnace slag by introducing CaCl_2_ and NaCl. Constr. Build. Mater..

[9] Yao J., Chen Q., Zeng L., Ding W. (2024). Preparation of calcium carbonate with microstructure and nanostructure from carbide slag for CO_2_ sequestration by using recyclable ammonium chloride. Particuology.

[10] Zhang H., He Y., Wang C., Guan Y., Ge Z., Sun R., Ling Y., Savija B. (2022). Statistical mixture design for carbide residue activated blast furnace slag foamed lightweight concrete. Constr. Build. Mater..

[11] Feng Y., Du Y., Zhou A., Zhang M., Li J., Zhou S., Xia W. (2021). Geoenvironmental properties of industrially contaminated site soil solidified/stabilized with a sustainable by-product-based binder. Sci. Total Environ..

[12] Yang J., Han C., Liu Y., Yan X., Dong S., Ma L., Dai Q., Huang B., Sun M., Yin X. (2024). CO_2_ capture by the slag from lignite’s chemical looping gasification using carbide slag. Energy.

[13] Zhang T., Chu G., Lyu J., Cao Y., Xu W., Ma K., Song L., Yue H., Liang B. (2022). CO_2_ mineralization of carbide slag for the production of light calcium carbonates. Chin. J. Chem. Eng..

[14] Jiang Q., He Y.M., Wu Y.L., Dian B., Zhang J.L., Li T.G., Jiang M. (2022). Solidification/stabilization of soil heavy metals by alkaline industrial wastes: A critical review. Environ. Pollut..

[15] Hao C., Deng M., Mo L., Liu K. (2012). Surface modification of fly ashes with carbide slag and its effect on compressive strength and autogenous shrinkage of blended cement pastes. J. Wuhan Univ. Technol.—Mater. Sci. Ed..

[16] Hanjitsuwan S., Phoo-ngernkham T., Li Ly Damrongwiriyanupap N., Chindaprasirt P. (2018). Strength development and durability of alkali-activated fly ash mortar with calcium carbide residue as additive. Constr. Build. Mater..

[17] Wang Y., Xu L., He X., Su Y., Miao W., Strnadel B., Huang X. (2022). Hydration and rheology of activated ultra-fine ground granulated blast furnace slag with carbide slag and anhydrous phosphogypsum. Cem. Concr. Compos..

[18] Zhao Z., Wu F., Dong S., Zhang Q., Huang C., Chen L. (2024). A low-carbon composite cementitious material manufactured by a combined process of red mud. Buildings.

[19] Lyu Z., Li Y., Fan M., Huang Y., Li C. (2024). Analysis of calcined red mud properties and related mortar performances. Fluid Dyn. Mater. Process..

[20] Sun Y.F., Dong F.Z., Liu J.T. (2009). Technology for recovering iron from red mud by Bayer process. Metrop. Mine.

[21] Zheng X.F. (2010). Recycling technology of aluminum and sodium from low temperature bayer progress red mud. Shandong Metall..

[22] Chen X.H., Chen Y., Gan M., Xu K.X. (2010). Precipitation and separation of vanadium from bayer process sodium aluminate solution. Chin. J. Process Eng..

[23] Pei J., Pan X., Zhang Y., Yu H., Tu G. (2021). A novel process to fully utilize red mud based on low-calcium sintering. J. Environ. Chem. Eng..

[24] Vangelatos I., Angelopoulos G.N., Boufounos D. (2009). Utilization of ferroalumina as raw material in the production of Ordinary Portland Cement. J. Hazard. Mater..

[25] Shao L., Wei G., Wang Y., Li Z., Zhang L., Zhao S., Zhou M. (2016). Preparation and application of acidified/calcined red mud catalyst for catalytic degradation of butyl xanthate in Fenton-like process. Environ. Sci. Pollut. Res..

[26] Vaclavikova M., Misaelides P., Gallios G., Jakabsky S., Hredzak S. (2005). Removal of cadmium, zinc, copper and lead by red mud, an iron oxides containing hydrometallurgical waste. Stud. Surf. Sci. Catal..

[27] Ciccu R., Ghiani M., Serci A., Fadda S., Peretti R., Zucca A. (2003). Heavy metal immobilization in the mining-contaminated soils using various industrial wastes. Miner. Eng..

[28] Lu G.Z., Zhang T.A., Bao L., Dou Z.H., Zhang W.G. (2008). Roasting pretreatment of high-sulfur bauxite. Chin. J. Process Eng..

[29] Samal S., Ray A.K., Bandopadhyay A. (2013). Proposal for resources, utilization, and processes of red mud in India-a review. Int. J. Miner. Process.

[30] Babisk M.P., Amaral L.F., Ribeiro L.S., Vieira CM F., Prado U.S., Gadioli MC B., Oliveira M.S., Luz F.S., Monteiro S.N., Filho F.C.G. (2020). Evaluation and application of sintered red mud and its incorporated clay ceramics as materials for building construction. J. Mater. Res. Technol..

[31] Alams S., Das S.K., Rao B.H. (2017). Characterization of coarse fraction of red mud as a civil engineering construction material. J. Clean. Prod..

[32] Ribeiro L.S., Babisk M.P., Prado U.S., Monteiro S.N., Vieira C.M.F. (2015). Incorporation of in natura and calcined red muds into clau ceramic. Mater. Res..

[33] Zhang D., Ghouleh Z., Shao Y. (2017). Review on carbonation curing of cement-based materials. J. CO_2_ Util..

[34] Zhang D., Ellis B.R., Jaworska B., Hu W.H., Li V.C. (2021). Carbonation curing for precast Engineered Cementitious Composites. Constr. Build. Mater..

[35] Chen T., Bai M., Gao X. (2021). Carbonation curing of cement mortars incorporating carbonated fly ash for performance improvement and CO_2_ sequestration. J. CO_2_ Util..

[36] Wang X., Zhan Q., Zhang X., Su Y., Zhou J. (2024). Study on improving the carbon sequestration properties of sintered red mud by regulating pressure. J. Build. Eng..

[37] (2023). Common Portland Cement.

[38] Song B., Hu X., Liu S., Shi C. (2022). Chloride binding of early CO_2_-cured Portland cement-fly ash-GGBS ternary pastes. Cem. Concr. Compos..

[39] (2021). Test Method of Cement Mortar Strength (ISO Method).

[40] Zhang Q., Dong S., Wu F., Cai Y., Xie L., Huang C., Zhao J., Yang S., Xu F., Zhu Z. (2024). Investigation of the macro performance and mechanism of biochar modified ultra-high performance concrete. Case Stud. Constr. Mater..

[41] Gupta S., Kashani A., Mahmood A.H., Han T. (2021). Carbon sequestration in cementitious composites using biochar and fly ash–Effect on mechanical and durability properties. Constr. Build. Mater..

[42] Yu H., Tang C., Mehdizadeh H., Guo M.Z., Ling T.C. (2024). Effects of early hydration of alite and belite phases on subsequent accelerated carbonation. Constr. Build. Mater..

[43] Li X., Ling T.C. (2020). Instant CO_2_ curing for dry-mix pressed cement samples: Consideration of CO_2_ concentrations coupled with further water curing. J. CO_2_ Util..

[44] Wang W., Zhou H., Li J., Tao F., Li C., Qian B., Jiang P., Jian P. (2021). Influence of carbonization process on the mechanical properties of Nano-MgO modified cement soil. Sustainability.

[45] Anjaneya D., Verma A., Pang S.D. (2021). Dual waste utilization in ultra-high performance concrete using biochar and marine clay. Cem. Concr. Compos..

[46] Norman M.D.A., Ferreira S.A., Jowett G.M., Bozec L., Gentleman E. (2021). Measuring the elastic modulus of soft culture surfaces and three-dimensional hydrogels using atomic force microscopy. Nat. Protoc..

[47] Christopher S., Rostislav C., Chao J., Josef H. (2023). Experimental investigations on early crack development in fully restrained reinforced concrete members with active zero-displacement control. Mater. Struct..

[48] Revathy T.D.R., Palanivelu K., Ramachandran A. (2017). Sequestration of carbon dioxide by red mud through direct mineral carbonation at room temperature. Glob. Warm..

[49] Bonenfant D., Kharoune L., Sauve S., Hausler R., Niquette P., Mimeault M., Kharoune M. (2008). CO_2_ sequestration by aqueous red mud carbonation at ambient pressure and temperature. Eng. Chem. Res..

[50] Yadav V.S., Prasad M., Khan J., Amritphale S.S., Singh M., Raju C.B. (2010). Sequestration of carbon dioxide (CO_2_) using red mud. Hazard. Mater..

[51] Sahu R.C., Patel R.K., Ray B.C. (2010). Neutralization of red mud using CO_2_ sequestration Cycle. Hazard. Mater..

[52] Ho H.J., Lizuka A., Shibata E. (2021). Utilization of low-calcium fly ash via direct aqueous carbonation with a low-energy input: Determination of carbonation reaction and evaluation of the potential for CO_2_ sequestration and utilization. Environ. Manag..

[53] Ukwattage N.L., Ranjith P.G., Wang S.H. (2013). Investigation of the potential of coal combustion fly ash for mineral sequestration of CO_2_ by accelerated carbonation. Energy.

[54] Montes-Hernandez G., Perez-Lopez R., Renard F., Nieto J.M., Charlet L. (2009). Mineral sequestration of CO_2_ by aqueous carbonation of coal combustion fly ash. Hazard. Mater..

[55] Uliasz-Bochenczyk A., EMokrzycki Piotrowski Z. (2009). Estimation of CO_2_ sequestration potential via mineral carbonation in fly ash from lignite combustion in Poland. Energy Proc..

[56] He L.L., Yu D.X., Lv W.Z., Wu J.Q., Xu M.H. (2013). A novel method for CO_2_ sequestration via indirect carbonation of coal fly ash. Eng. Chem. Res..

